# Statistical modeling of the effect of rainfall flushing on dengue transmission in Singapore

**DOI:** 10.1371/journal.pntd.0006935

**Published:** 2018-12-06

**Authors:** Corey M. Benedum, Osama M. E. Seidahmed, Elfatih A. B. Eltahir, Natasha Markuzon

**Affiliations:** 1 Draper, Cambridge, Massachusetts, United States of America; 2 Department of Epidemiology, Boston University School of Public Health, Boston, Massachusetts, United States of America; 3 Ralph M Parsons Laboratory, Massachusetts Institute of Technology, Cambridge, Massachusetts, United States of America; University of Washington, UNITED STATES

## Abstract

**Background:**

Rainfall patterns are one of the main drivers of dengue transmission as mosquitoes require standing water to reproduce. However, excess rainfall can be disruptive to the *Aedes* reproductive cycle by “flushing out” aquatic stages from breeding sites. We developed models to predict the occurrence of such “flushing” events from rainfall data and to evaluate the effect of flushing on dengue outbreak risk in Singapore between 2000 and 2016.

**Methods:**

We used machine learning and regression models to predict days with “flushing” in the dataset based on entomological and corresponding rainfall observations collected in Singapore. We used a distributed lag nonlinear logistic regression model to estimate the association between the number of flushing events per week and the risk of a dengue outbreak.

**Results:**

Days with flushing were identified through the developed logistic regression model based on entomological data (test set accuracy = 92%). Predictions were based upon the aggregate number of thresholds indicating unusually rainy conditions over multiple weeks. We observed a statistically significant reduction in dengue outbreak risk one to six weeks after flushing events occurred. For weeks with five or more flushing events, compared with weeks with no flushing events, the risk of a dengue outbreak in the subsequent weeks was reduced by 16% to 70%.

**Conclusions:**

We have developed a high accuracy predictive model associating temporal rainfall patterns with flushing conditions. Using predicted flushing events, we have demonstrated a statistically significant reduction in dengue outbreak risk following flushing, with the time lag well aligned with time of mosquito development from larvae and infection transmission. Vector control programs should consider the effects of hydrological conditions in endemic areas on dengue transmission.

## Author summary

Dengue transmission is sensitive to fluctuations in rainfall and other weather conditions because it is transmitted by the *Aedes* mosquito. Recent studies have identified that extreme rainfall can result in mosquito breeding site flushing. However, these rainfall conditions have neither been described nor evaluated for their potential effect on dengue transmission. In this study, we applied machine learning and regression approaches to identify rainfall thresholds associated with mosquito breeding site flushing in Singapore. We then estimated the association between the number of flushing events per week and dengue outbreaks in the following weeks. Here we demonstrate that flushing events are accurately predicted by historical rainfall patterns. We also show that flushing events are associated with a statistically significant reduction in dengue outbreak risk up to six weeks after the flushing events occurred. This research suggests that dengue predictive and early warning systems must consider hydrological conditions and other contributing factors to accurately predict near-term dengue risk.

## Introduction

Dengue, a disease transmitted by *Aedes* mosquitoes, is a global public health problem. Dengue is endemic in more than 100 tropical and subtropical countries where 30–50% of the global population is at risk for infection [1–4]. Annually, there are an estimated 50–100 million cases of dengue, with 500,000 of these cases developing into life-threatening Dengue Hemorrhagic Fever and Dengue Shock Syndrome [5].

Various weather factors influence dengue incidence. Temperature and humidity impact dengue incidence by affecting adult feeding behavior, larvae development, and mosquito survival [2,5–16]. Small increases in average monthly temperature (e.g., 1°C) have been associated with a considerable increase in dengue incidence, leading to a 45% increase in the number of cases in subsequent months in Brazil and China [4,5,17–30].

Although mosquitoes require sufficient rainfall for breeding and larval development [2,7–9,31,32], too much rainfall can be detrimental [33–35]. Excessive rainfall can cause breeding sites to overflow, disrupting mosquito breeding and destroying developing larvae. Mosquito breeding site “flushing”, where water levels exceed a breeding site’s drainage threshold and wash away mosquito larvae, has been observed in both experimental and field settings [33–36]. In experimental studies, simulated heavy rainfall washed away the majority of mosquito larvae and resulted in significant larvae mortality [33,35,36]. The extent of the effect of flushing depended on rainfall intensity, container size, and larvae age [33,35]. A field study in Singapore demonstrated that dengue incidence is lowest following months where flushing events are most frequent suggesting that flushing events may influence how and when dengue transmission occurs [34].

Multiple studies have described the weather drivers of dengue incidence using regression and time series models [4,5,10,17–30,37–39]. While relationships between temperature and humidity and dengue incidence have been consistent, the relationship between rainfall and dengue incidence has remained unclear. Associations between rainfall and dengue incidence have ranged from weak or no connection [19,25,27–29,39], to as much as a 21% increase in dengue incidence in response to increased rainfall [10,19,26,37]. Machine learning tools have been used to predict the occurrence of dengue based on a combination of weather parameters, including rainfall [40–48]. Though successful in predicting weekly and monthly dengue incidence with over 90% accuracy, these models did not explain the relationship between rainfall and dengue.

One interesting and still unanswered question relates to the influence of flushing on dengue incidence. Observations by Seidahmed and Eltahir [34] of larvae survival in storm drains in Singapore following rainfall events have confirmed that flushing mostly occurs during the Northeast monsoon season. The authors noted that the season with the highest rainfall levels and most flushing events preceded the season with traditionally low dengue incidence. However, the study did not quantify the rainfall patterns leading to flushing nor its effect on dengue spread.

This paper proposes a quantitative approach associating flushing, expressed through rainfall patterns, with the subsequent fluctuations in dengue outbreak risk in Singapore between 2000 and 2016. The methodology uses entomological data from Singapore [34] combined with historical rainfall data to quantify the rainfall conditions associated with mosquito larvae wash-out, or “flushing”. A regression model is then used to estimate the effect of flushing events on dengue outbreaks. We show a statistically significant 16–70% reduction in dengue outbreak risk in one to six weeks following flushing events.

## Materials and methods

### Study area

Singapore is located on the southern-most tip of the Malay Peninsula with a population of 5.6-million people [49]. Dengue is hyper-endemic, where serotypes I-IV co-circulate, and is transmitted year-round, with peak incidence occurring between July and September [50–53]. Singapore has a tropical rainforest climate (Köppen: *Af*) with two monsoon seasons, the Northeast and Southwest monsoons. The former is associated with heavy rainfall between November and March while the latter occurs between June and October and is relatively drier [54]. Average annual precipitation is nearly 2.3 meters. Average daily temperature is stable throughout the year, where average daily temperatures of the hottest and coolest months differ by 1.9°C.

### Surveillance data

Weekly dengue case counts in Singapore from 2000–2016 (N = 887) were obtained from the Weekly Infectious Disease Bulletin of the Singapore Ministry of Health [55]. Confirmed cases were reported by all public and private hospitals and laboratories who are mandated to report all clinically and lab diagnosed cases of dengue within 24 hours [56,57].

### Entomological data

Longitudinal entomological surveys were obtained from a previously published work of Seidahmed and Eltahir [34]. These surveys were conducted in the Geylang neighborhood of Singapore, a highly urbanized neighborhood located east of the Singapore River [34]. Geylang is classified as being hyperendemic for dengue by the National Environmental Agency since dengue transmission occurs year-round [34]. Geylang is estimated to have a resident population of 32,000 and an even larger non-resident population which is a result of the large amount of cheap housing options that are primarily used by foreign laborers [34].

During entomological data collection, random aquatic surveys were completed twice a week between August 2014 and August 2015 (except between February 21st and March 10th), resulting in 107 days of entomological observations. For each survey, trained inspectors examined all potential outdoor natural and artificial mosquito breeding sites (e.g., open and closed roadside storm drains and non-drain sites such as canvas sheets, pails and flowerpots), looking for mosquito aquatic stages in randomly selected neighborhood blocks. Samples of aquatic stages were taken and evaluated for taxonomic classification. A subsample of aquatic specimens was retained until adult emergence to confirm taxonomic identification. Taxonomic keys [58–60] were used to classify sampled aquatic stages and emerged adults [34].

A total of 6,824 samples were taken from potential breeding sites (5,818 samples from open and closed storm drains) [34]. Sixty-seven breeding sites (53 occurring in storm drains) were positive for *Ae*. *aegypti* breeding [34]. Particular attention was then given to the 53 positive *Ae*. *aegypti* breeding sites that were found in open and closed storm drains [34]. Breeding sites of *Ae*. *Aegypti* were mainly found in the southern part of Geylang where denser urban drainage network and low-rise housing predominate [61].

**Fig 1:**
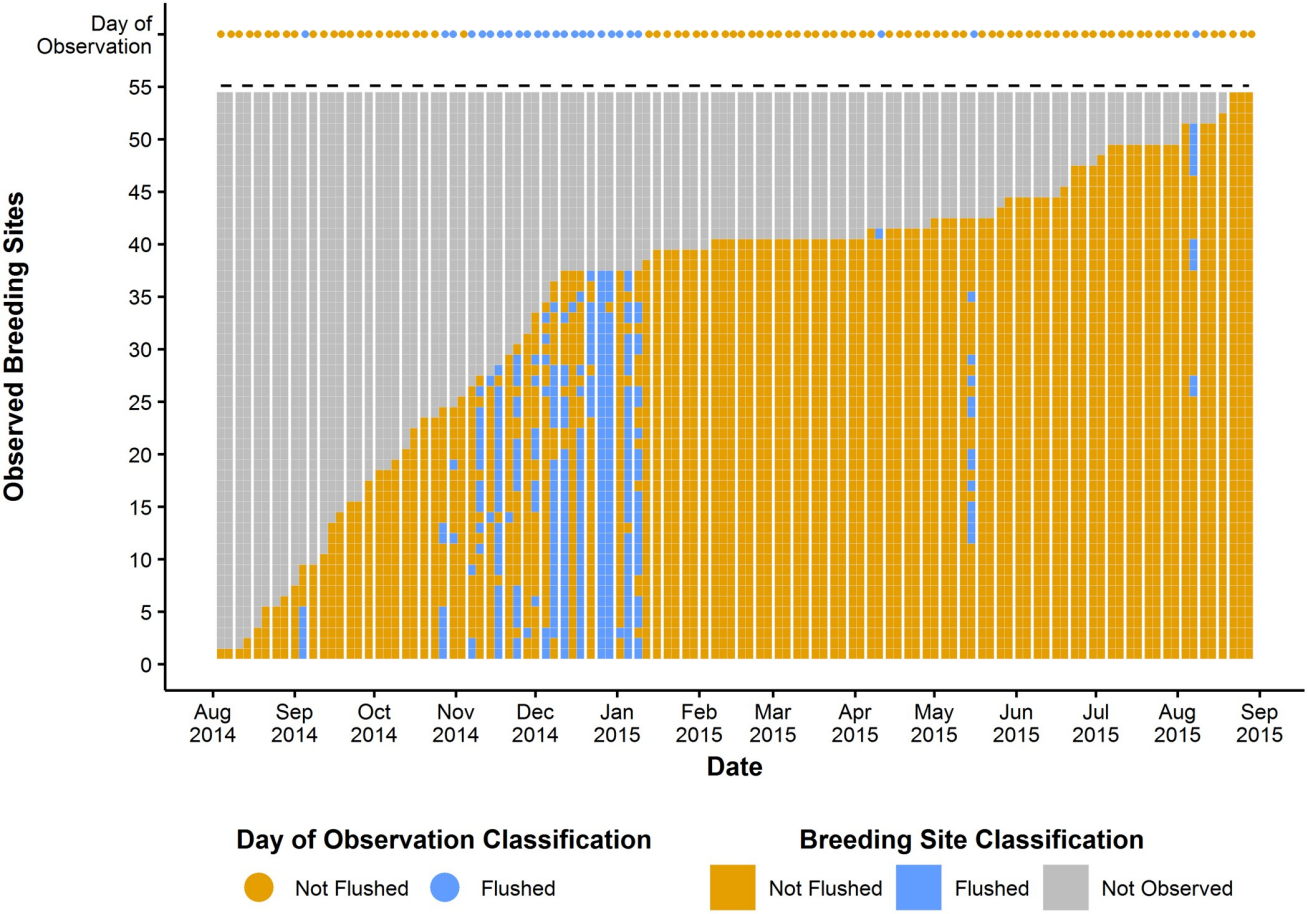
Timeline of observed *Ae*. *aegypti* breeding sites in Geylang, Singapore, August 2014 –August 2015. There were 107 days of entomological observations where 53 breeding sites, once identified as positive for *Ae*. *aegypti* breeding, were monitored for changes in hydrological conditions. Breeding sites where water exceeded the drainage threshold were classified as “Flushed”. For each day of observation, if at least one breeding site was observed as flushed, the day of observation was defined as flushed. If no breeding sites were observed as flushed then the day of observation was classified as not flushed. This figure, “Timeline of observed *Ae*. *aegypti* breeding sites in Geylang, Singapore, August 2014 –August 2015 is a derivative of “Timeline of the breeding drains of *Aedes aegypti* in Geylang, Singapore: August 2014 –August 2015” by Seidahmed and Eltahir [34], used under CC BY.

The 53 positive breeding sites occurring in storm drains were continuously monitored for hydrological conditions and changes in the presence of mosquito larvae. For each visit during the monitoring phase, the following four conditions were observed: 1) stagnant water and positive for aquatic stages, 2) stagnant water and negative for aquatic stages, 3) dry and negative for aquatic stages, and 4) flushed and negative for aquatic stages [34]. No sites were identified as flushed and positive for aquatic stages or dry and positive for aquatic stages. We then classified each day of observation as **“***Flushed*” if at least one breeding site was classified as “flushed and negative”, meaning that water had exceeded the drainage threshold (indicated by the storm drain overflowing, or evidence of an overflow) for the breeding site and mosquito larvae were not present. Of the 107 days of entomological observations, 25 were classified as flushed and 82 as non-flushed (Fig 1). The majority of flushing events (84%) occurred during the Northeast monsoon, while only 23% of non-flushing events happened during this time. This data was used to develop a model to predict flushing occurrence for the entire study period.

### Weather data

Daily weather data, including rainfall and temperature, were obtained from Tanjong Katong weather station (selected for its proximity to Geylang) [62]. Humidity data were extracted from remote sensed reanalysis data [63] and El Niño Southern Oscillation (ENSO) data were obtained from the Climate Prediction Center [64], both of which are operated by the National Oceanic and Atmospheric Administration. The ENSO index used was the normalized Oceanic Niño Index for Niño region 3.4 [65–68] and used to define the ENSO phase (i.e., El Niño, La Niña, Neutral). Weather data were obtained from 1/1/1999–12/31/2016. Missing weather data were imputed using multiple imputations through chained equations using the *MICE* R package [69].

### Exposure assessment: Modeling and predicting flushing event occurrence

Entomological data detailing the hydrological conditions of breeding sites in Singapore were only available for a single year (August 2014 –August 2015) during the study period (2000–2016). We developed the **Predictive fLUshing-Mosquito model** (PLUM) which predicts the occurrence of flushing events based on the temporal variation in daily rainfall over several weeks preceding the day of interest and allows the extension of the prediction to dates when no entomological observations are available. The objective was not only to make a daily prediction but to identify more general ‘flushing’ conditions leading to drains’ overflow. Early flooding warning systems use rainfall thresholds to predict flooding occurrence [70]. The PLUM model operates in a similar fashion by identifying a set of variables and their thresholds associated with flushing occurrence.

The PLUM model was developed using the entomological observations and the corresponding rainfall data between August 2014 to August 2015. The general model framework can be found in S1 Fig. The proposed approach identifies rainfall thresholds associated with a higher likelihood of flushing occurrence. We used supervised machine learning models to associate the identified thresholds with flushing occurrence. Each model was trained using a balanced training set where non-flushed observations were randomly under-sampled to generate a 1:1 ratio of flushed to non-flushed observations in the training set to prevent the model from classifying all observations as the majority class [71]. Each model was evaluated on unseen data using leave-one-out cross validation. The PLUM model was then extended to the entire study period, to predict the occurrence of flushing events in Singapore between 2000 and 2016.

#### Data preprocessing

The PLUM model operates on a daily basis, with an outcome, **“***flushing***”,** defined as a day with at least one flushed breeding site recorded in the entomological data. Rainfall variables were created to summarize trends in rainfall. We created 38 time-varying rainfall variables from the weeks preceding the day of interest reflecting frequency, intensity, and total rainfall. Rainfall was characterized using several different variables to capture the different mechanisms by which flushing may occur (i.e., very intense rainfall directly preceding the flushing event or elevated rainfall over several weeks followed by moderate rainfall directly preceding the flushing event). These variables were selected based upon their association with mosquito abundance and because they are conventionally used in studies of mosquito breeding ecology (Table 1) [36,72–76].

**Table 1:** Rainfall variables created for PLUM model development. These variables were created to characterize rainfall several different ways in order to capture the different mechanisms by which storm drains may be flushed.

Variables	Total number of variables created
**Daily rainfall variables**	
The number of rainy days in the last 7-day period	1 variable
Average daily rainfall per rainy day in the last 7, 14, 21, and 28-day period	4 variables
The ranked order (1^st^ highest, 2^nd^ highest,…, 7^th^ highest) daily total rainfall in the previous 7-day period	7 variables
Peak daily total rainfall in the previous 1-, 2-,…, 6-day period	6 variables
**Cumulative rainfall variables**	
Cumulative total rainfall covering a period of 1, 2, 3,…, 20 weeks prior	20 variables

#### Identifying thresholds associated with flushing occurrence

One of the objectives of the PLUM model was to identify flushing conditions expressed through the ‘thresholds’ of various combinations of rainfall parameters. We used the Univariate Flagging Algorithm (UFA) [64] to identify conditions leading to flushing.

UFA is a threshold detection algorithm that identifies an optimal cutpoint for a single continuous variable (e.g., 1-week cumulative rainfall) that is associated with a statistically significant higher (“high risk”) likelihood of the outcome (flushing). The algorithm evaluates candidate thresholds along the variable space, selecting the threshold that optimizes the difference in the outcome rate for observations that fall outside of the threshold and a baseline rate. The baseline rate is defined as the outcome rate within the interquartile range of the evaluated variable. Two such thresholds can be identified, one above and one below the median. As recommended by the algorithm developers, statistically significant thresholds were identified using a p-value of 0.01. A total of 36 high risk thresholds were identified. The identified flushing thresholds are specific to storm drains that are similar to the sampled storm drains (see section 2.3 *Entomological Data*) and are invariant to time because the hydrological characteristics and drainage thresholds will remain constant unless the storm drains are rebuilt using new dimensions, are damaged, or are obstructed by accumulated rubbish or sediment.

#### PLUM model development and evaluation

The PLUM model predicts daily flushing occurrence based upon two variables, the aggregate number of high risk thresholds that were met per day for both cumulative and daily rainfall variables (all models that were investigated are described in *section 1*.*1* of S1 Text). The general formula for the PLUM model is given as follows:

$$Flushed_{D}=\beta_{1}(Thresholds\;Met_{D,Daily-High\;Risk})+\beta_{2}\left(Thresholds\;Met_{D,Cumulative-High\;RIsk}\right)+\beta_{0}$$ (1)

Where *Flushed*_*D*_ is the flushing status for day D; *Thresholds Met*_*D*, *Daily-High Risk*_ is the number of high risk daily rainfall thresholds met for day D; *Thresholds Met*_*D*, *Cumulative-High Risk*_ is the number of high risk cumulative rainfall thresholds met for day D; β_1_ and β_2_ are the model coefficients; and β_0_ is the model intercept. This model used a classification threshold of 0.52, meaning that when predicted values where greater than or equal to 0.52, the model predicted “flushed”, otherwise the model predicted “not flushed”.

We performed a sensitivity analysis to evaluate how different definitions of the outcome variable “*flushing*” affected PLUM model performance. The sensitivity analysis is described in detail in *section 1*.*2* of S1 Text.

#### Modeling the association between flushing events and dengue outbreaks

We developed a distributed lag nonlinear logistic regression model [77] to estimate the effect of the number of flushing events per week on the risk of dengue outbreak occurrence, controlling for potential confounders. The model is given as follows:

$$Outbreak_{w}=\Sigma_{l=1}^{l=20}ns(Weekly\;Flushing_{w-l},\;3,\;3)+\Sigma_{i=1}^{i=20}Temp_{w-i}+\Sigma_{j=1}^{j=20}AH_{w-j}+Outbreak_{w-1}+Monsoon_{w}+ENSO_{w}$$ (2)

Where *Outbreak*_*w*_ is a binary variable reflecting outbreak status during week *W*; *Weekly flushing*_*w-l*_ is the number of flushing events per week predicted by the PLUM model for week *W-l* (l [1-20]); *Temp*_*w-i*_ is the average weekly temperature for week *W-i* (i [1-20]); *AH*_*w-j*_ is the average weekly absolute humidity for week *W-j* (j [1-20]) and was estimated using the approach described by Xu et al. [5]; *Outbreak*_*w-1*_ is a binary variable reflecting the outbreak status during week *W-1*; *Monsoon*_*w*_ is the season (Northeast monsoon, Southwest monsoon, Non-monsoon) for week *W*; and *ENSO*_*w*_ is the current ENSO period (El Niño, La Niña, Neutral) for week *W*.

We define the outcome, *Outbreak*_*w*_, as follows:

$$Outbreak_{w}\{\begin{array}{ll} 1, & Incidence_{w}\geq Outbreak\;threshold_{year}\\ 0, & Otherwise \end{array}\prime$$ (3)

where the outbreak threshold for each year is defined as:

$$Outbreak\;threshold_{year}=mean{\left(weekly\;incidence\right)}_{year}+\;standard\;deviation{\left(weekly\;incidence\right)}_{year}$$ (4)

The exposure of interest, “*Weekly flushing*_*w*_” was created by predicting whether or not a flushing event occurred for each day in the study period with the PLUM model and aggregating the number of predicted flushing events per week. In Eq (2), a natural cubic spline function was used to model the nonlinear weekly flushing association with 3 degrees of freedom and the lagged association using 3 degrees of freedom.

Potential confounders and modifiers, average weekly temperature, average weekly absolute humidity, monsoon season, ENSO phase, and the previous week’s outbreak status, were selected based upon their known relationship with rainfall or dengue [2,5–16,31,32,78–80]. Data for these variables were obtained from the weather and surveillance data sets. We utilized a simple confounder model which assumed a linear relationship between each confounder and dengue outbreak risk. A simple confounder model was selected because we are interested in the relationship between flushing and dengue outbreak risk and model estimates are relatively insensitive to the choice of confounder model [81]. In this analysis, we included a lag period of one to 20 weeks for the weekly flushing, temperature and humidity variables. This period was selected based upon a literature review on the effects of weather factors on vectors and dengue transmission [51,82–88].

## Results

### Rainfall conditions associated with flushing events

We applied UFA to identify thresholds associated flushing. Running UFA for all 38 rainfall variables, we identified 36 thresholds (20 for cumulative rainfall variables, 16 for daily rainfall variables) associated with an increased likelihood of flushing occurrence. The likelihood of flushing occurrence was found to increase when variable values were greater than or equal to the associated high risk thresholds. All UFA identified thresholds can be found in S1 Table.

### Predicting flushing events from rainfall pattern

In total, there were 107 days of entomological observations, 25 were defined as flushed and 82 were defined as not flushed. We used the PLUM model (described in *section 2*.*5*) to classify each entomological observation using leave-one-out cross validation. The PLUM model made predictions based upon two variables, the aggregate number of high risk thresholds that were met per day for both cumulative and daily rainfall variables. The PLUM model achieved 92% accuracy and demonstrated a strong ability to discriminate between flushed and non-flushed observations (Table 2).

**Table 2:** Performance of PLUM model classification on the unseen test data. The model used the aggregate number of high risk thresholds that were met per day for both cumulative and daily rainfall variables. Evaluation measures include F1-score (F1), accuracy (Acc), positive predictive value (PPV), Sensitivity (Se), area under the receiver operating characteristics curve (AUC), specificity (Sp), negative predictive value (NPV).

Model	Variables	Acc	F1	PPV	Se	AUC	Sp	NPV
Logistic	# cumulative rainfall high risk thresholds # daily rainfall high risk thresholds	92%	0.84	0.77	0.92	0.94	0.91	0.97

**Fig 2:**
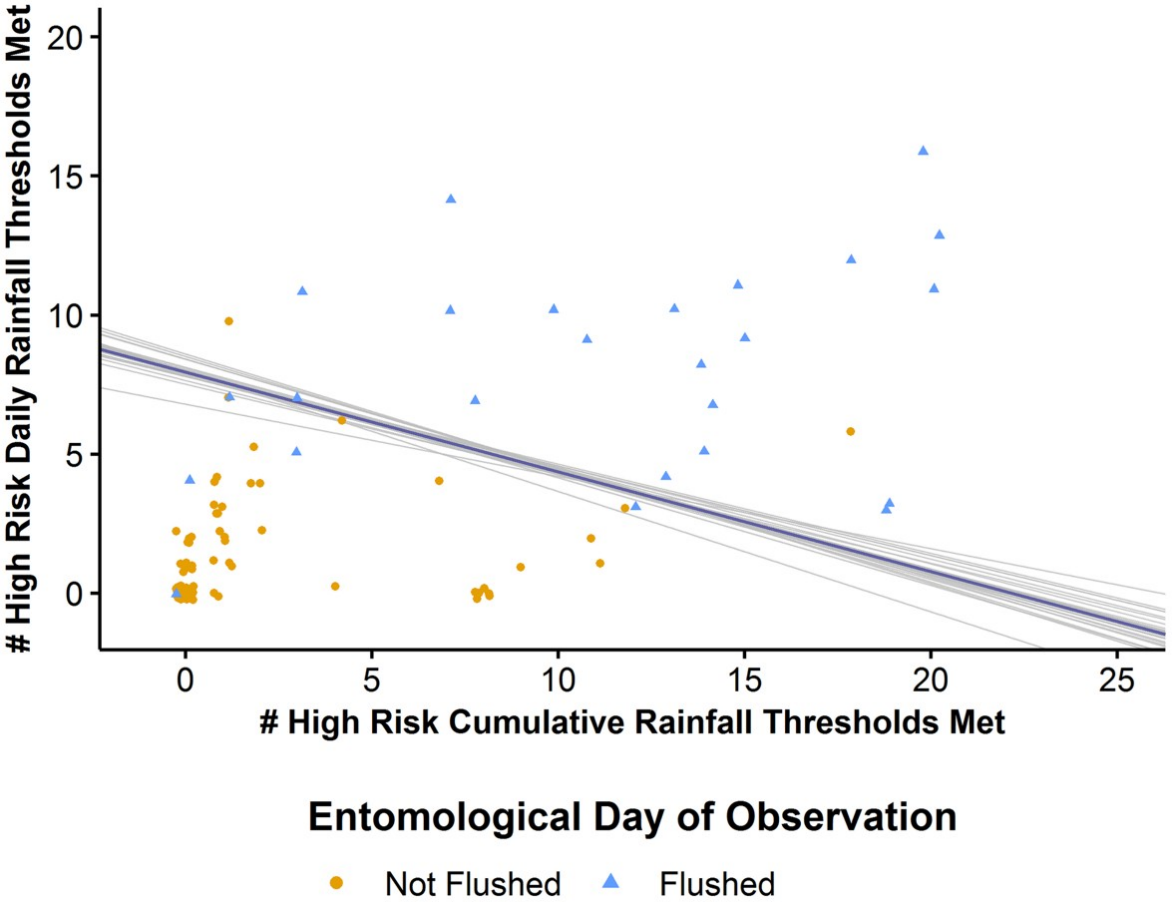
Results of PLUM model associating flushing with the number of high risk thresholds met for cumulative and daily rainfall variables. There is a clearly defined threshold that almost perfectly separates flushed (blue) and non-flushed (orange) observations based upon the aggregate number of high risk thresholds that were met per day for both cumulative and daily rainfall variables. Each gray line represents Eq (1) fit to each leave-one-out cross validation sample while the blue line represents the mean fit from all of the leave-one-out cross validation samples.

There is a well-defined threshold resulting in nearly perfect separation between flushed and non-flushed observations (Fig 2). We fit Eq (1) to each cross validation sample and identified the mean value for each model coefficient resulting in the following model:

$$Flushed_{D}=0.56(Thresholds\;Met_{D,Daily-\;High\;Risk})+0.20\left(Thresholds\;Met_{D,Cumulative-High\;RIsk}\right)+7.94.$$ (5)

We observed that the fit of Eq (1) for each cross validation sample was stable indicating good generalizability of the PLUM model.

We extended the PLUM model to the entire study period. Using Eq (5) we assigned a value indicating whether or not flushing occurred on each day within the study period. For the study period, 1,242 (21.2%) days were classified as flushing, a similar proportion to the number of flushed days (25, 23.4%) in the observed entomological data time period. These results were used in creating the “weekly flushing” variable which is an aggregator of daily flushing events per week.

### Measuring the association between flushing and dengue outbreaks

We used a distributed lag nonlinear logistic regression model to evaluate the association between flushing occurrence and the risk of a dengue outbreak in the weeks following the flushing events.

Dengue incidence was reported in 887 weeks between the years 2000 and 2016. Summary statistics for outbreak occurrence, flushing occurrence, and other weather variables during the study period are presented in Table 3. During the study period, 138 (15.6%) weeks were defined as an *Outbreak* week based upon the selected criteria. There is also evidence of seasonal variation in outbreak occurrence and rainfall. Outbreak weeks were at least three times as likely to occur during the Southwest monsoon compared with the Northeast and Non-monsoonal periods. Total weekly rainfall shows a seasonal pattern where weeks during the Northeast monsoon have on average 15-20mm more rainfall than other weeks. Due to increased rainfall, flushing events were most likely to occur during the Northeast monsoon as compared with any other season.

**Table 3:** Descriptive statistics for weekly data on weather variables and dengue outbreak occurrence in Singapore, 2000–2016.

Variable	Northeast monsoon	Non-monsoon	Southwest monsoon	All Weeks
**Number of total weeks**	368	148	371	887
Number of outbreak weeks	32 (8.7%)	3 (2.0%)	103 (27.8%)	138 (15.6%)
Total weekly rainfall (Mean ± SD)	52.6 (60.4)	38.2 (33.7)	33.6 (30.4)	42 (46.5)
Daily temperature (Mean ± SD)	27.2 (0.7)	28.4 (0.7)	28.1 (0.7)	27.8 (0.9)
Daily absolute humidity (Mean ± SD)	25.3 (1.5)	26.7 (1.0)	25.8 (1.2)	25.8 (1.4)
Number of flushing events per week (Mean ± SD)	2.3 (2.5)	1.2 (1.9)	0.6 (1.3)	1.4 (2.1)

Abbreviations: SD, standard deviation

**Fig 3:**
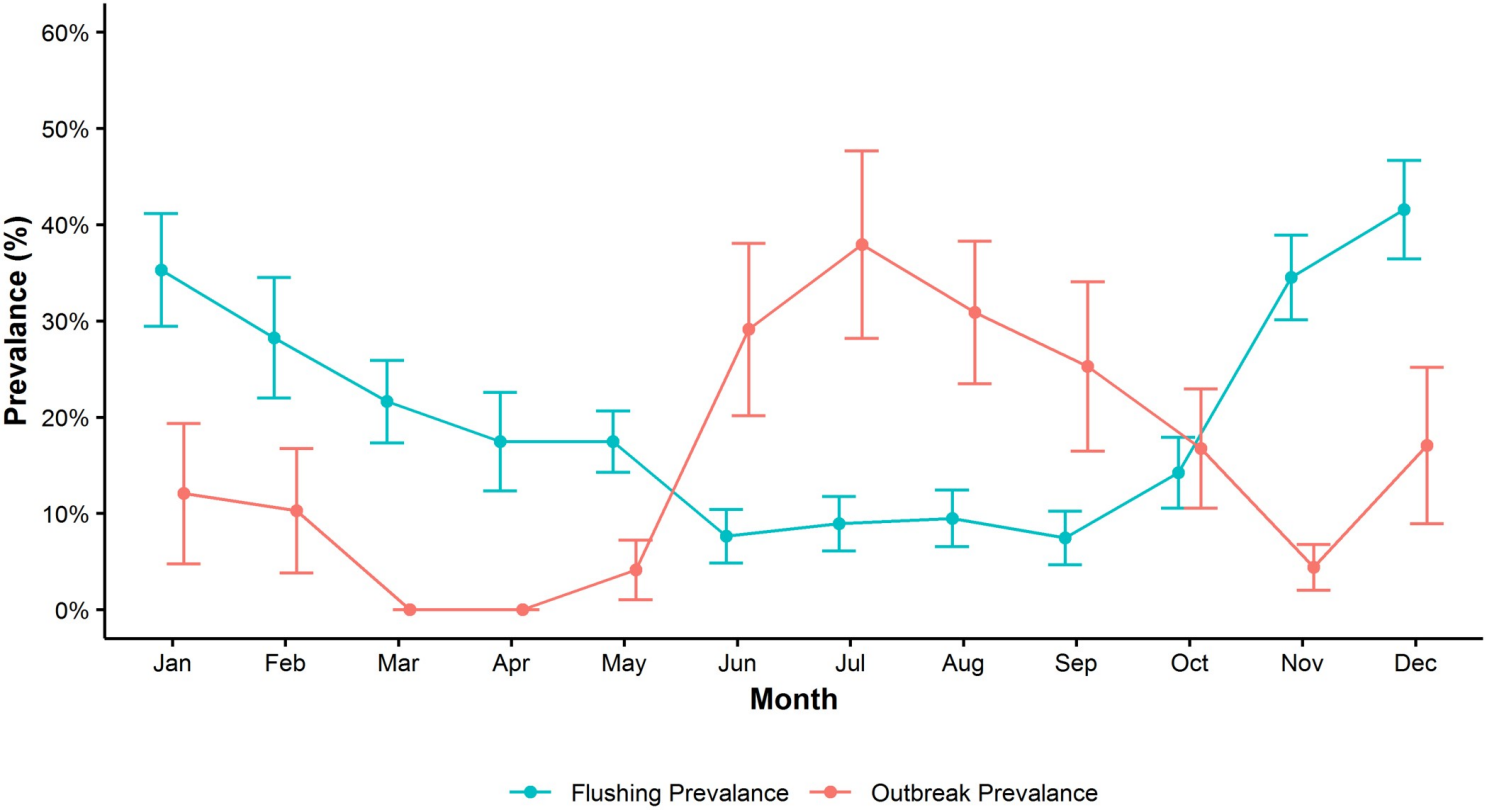
The average prevalence of flushing events and dengue outbreak weeks by month, Singapore, 2000–2016. In months where flushing event prevalence is highest (blue) dengue outbreak week prevalence (red) is lowest, and vice versa.

Fig 3 shows the prevalence of flushing events and the prevalence of outbreak weeks by month. Here we observe a negative association in which months where flushing event prevalence is highest (November to February) the outbreak week prevalence is low. Moreover, in months where flushing event prevalence is low (June to September), outbreak week prevalence is highest.

**Fig 4:**
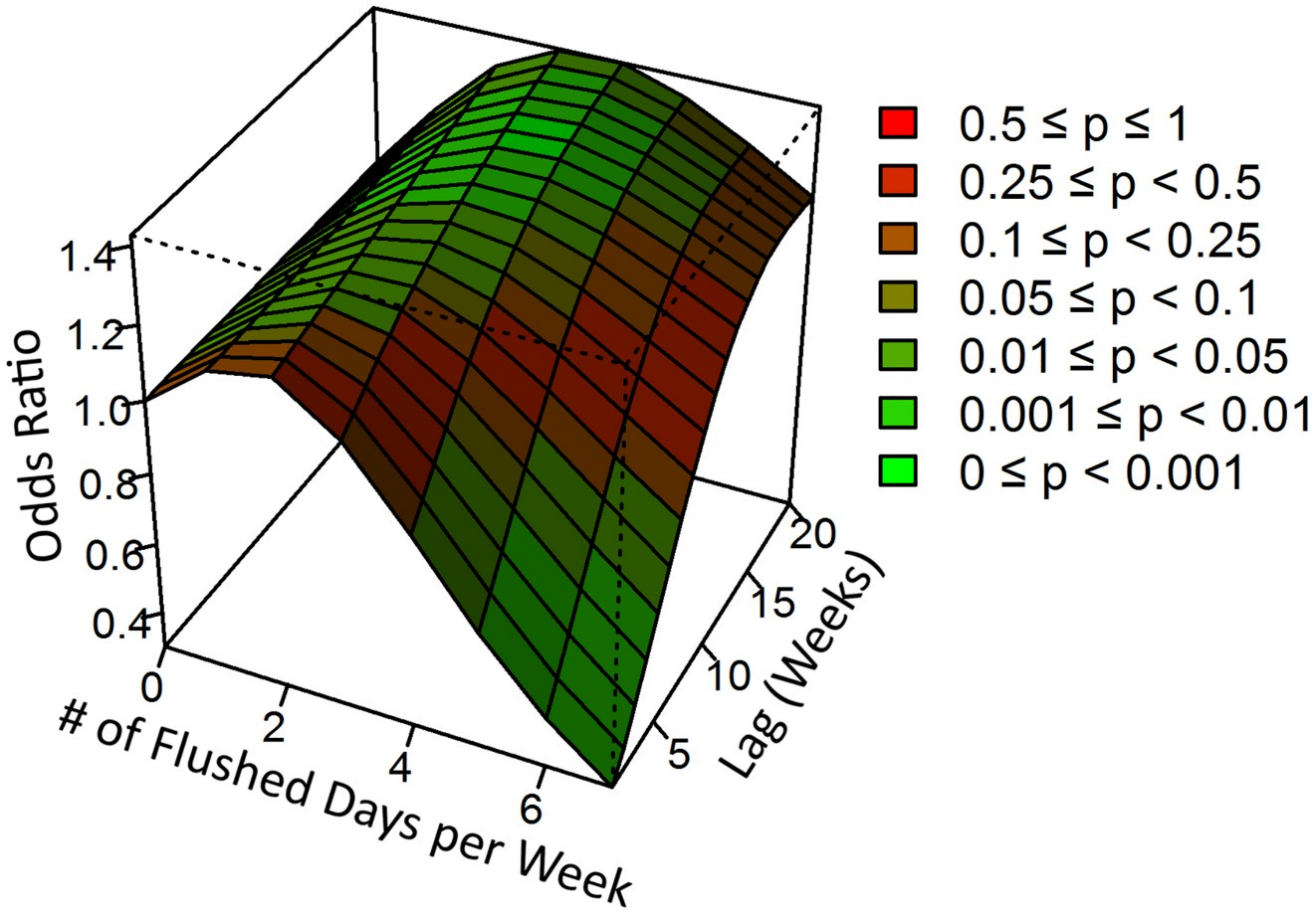
Association between number of flushing events per week and dengue outbreak occurrence over 20 lag weeks. The risk of an outbreak occurring within one to six weeks after the week of consideration was significantly lower if five or more flushing events occurred during the considered week.

Regression analysis, Eq (2), demonstrates a negative association between flushing events and dengue outbreak risk (Fig 4). We identified a nonlinear association between the number of flushing events per week and dengue outbreak risk that varied over the lag dimension. The risk of an outbreak occurring was significantly lower for weeks where five or more flushing events occurred compared with weeks with zero flushing events; this relationship remained significant up to six weeks after the flushing events occurred (Table 4). Weeks where seven flushing events occurred, there was between a 30–70% reduction in the risk of an outbreak up to six weeks after the flushing events occurred. Smaller reductions in dengue outbreak risk were also observed when five (16–38% reduction in risk up to four weeks after the flushing events) and six (24–56% reduction in risk up to five weeks after the flushing events) flushing events occurred in a week.

**Table 4:** Association between number of flushing events per week and dengue outbreak occurrence over 20 lag weeks. When five or more flushing events occurred in a week, compared with 0 flushing events, the risk of a dengue outbreak occurring in the subsequent weeks was significantly reduced up to six weeks after the flushing events occurred.

Weeks after flushing events	1 Day Flushed OR (95%CI)	2 Day Flushed OR (95%CI)	3 Day Flushed OR (95%CI)	4 Day Flushed OR (95%CI)	5 Day Flushed OR (95%CI)	6 Day Flushed OR (95%CI)	7 Day Flushed OR (95%CI)
1 Week	1.13 (0.88–1.44)	1.16 (0.77–1.74)	1.04 (0.67–1.62)	0.84 (0.54–1.30)	**0.62 (0.38–1.02)**	**0.44 (0.22–0.87)**	**0.30 (0.11–0.79)**
2 Weeks	1.13 (0.93–1.37)	1.17 (0.85–1.60)	1.07 (0.76–1.51)	0.89 (0.64–1.25)	**0.69 (0.47–1.02)**	**0.51 (0.29–0.88)**	**0.37 (0.17–0.80)**
3 Weeks	1.13 (0.97–1.30)	1.18 (0.93–1.49)	1.11 (0.86–1.43)	0.95 (0.74–1.22)	**0.76 (0.57–1.03)**	**0.59 (0.38–0.90)**	**0.44 (0.24–0.82)**
4 Weeks	**1.13 (1.00–1.27)**	**1.19 (0.98–1.44)**	1.14 (0.93–1.39)	1.01 (0.83–1.22)	**0.84 (0.67–1.05)**	**0.67 (0.48–0.94)**	**0.53 (0.32–0.86)**
5 Weeks	**1.13 (1.01–1.26)**	**1.20 (1.01–1.42)**	**1.17 (0.97–1.40)**	1.06 (0.89–1.25)	0.91 (0.75–1.10)	**0.76 (0.57–1.01)**	**0.62 (0.40–0.94)**
6 Weeks	**1.13 (1.01–1.26)**	**1.21 (1.01–1.44)**	**1.19 (0.99–1.44)**	1.11 (0.93–1.31)	0.98 (0.81–1.18)	0.84 (0.64–1.10)	**0.70 (0.47–1.05)**
7 Weeks	**1.13 (1.00–1.27)**	**1.22 (1.01–1.47)**	**1.22 (1.00–1.49)**	1.15 (0.96–1.39)	1.04 (0.86–1.27)	0.91 (0.69–1.20)	0.79 (0.53–1.19)
8 Weeks	**1.13 (1.00–1.28)**	**1.23 (1.00–1.50)**	**1.24 (1.00–1.54)**	**1.19 (0.98–1.46)**	1.10 (0.89–1.35)	0.99 (0.74–1.31)	0.87 (0.57–1.32)
9 Weeks	**1.14 (1.00–1.29)**	**1.24 (1.00–1.52)**	**1.26 (1.01–1.58)**	**1.23 (1.00–1.51)**	1.15 (0.93–1.43)	1.05 (0.78–1.41)	0.95 (0.62–1.45)
10 Weeks	**1.14 (1.00–1.29)**	**1.25 (1.01–1.53)**	**1.29 (1.03–1.61)**	**1.26 (1.02–1.56)**	1.20 (0.96–1.49)	1.11 (0.82–1.49)	1.01 (0.66–1.54)
11 Weeks	**1.14 (1.01–1.29)**	**1.26 (1.03–1.54)**	**1.30 (1.05–1.63)**	**1.29 (1.05–1.59)**	**1.24 (1.00–1.53)**	1.16 (0.87–1.54)	1.07 (0.71–1.61)
12 Weeks	**1.15 (1.02–1.29)**	**1.26 (1.04–1.54)**	**1.32 (1.07–1.63)**	**1.32 (1.08–1.61)**	**1.27 (1.03–1.57)**	1.20 (0.91–1.58)	1.11 (0.75–1.65)
13 Weeks	**1.15 (1.03–1.29)**	**1.27 (1.06–1.53)**	**1.34 (1.09–1.64)**	**1.34 (1.10–1.63)**	**1.30 (1.06–1.59)**	1.23 (0.94–1.60)	1.15 (0.80–1.66)
14 Weeks	**1.15 (1.03–1.29)**	**1.28 (1.07–1.54)**	**1.35 (1.11–1.65)**	**1.36 (1.12–1.65)**	**1.32 (1.08–1.60)**	**1.25 (0.98–1.61)**	1.17 (0.83–1.66)
15 Weeks	**1.16 (1.04–1.30)**	**1.29 (1.07–1.56)**	**1.36 (1.11–1.68)**	**1.37 (1.13–1.67)**	**1.34 (1.10–1.63)**	**1.27 (0.99–1.62)**	1.19 (0.85–1.66)
16 Weeks	**1.16 (1.03–1.31)**	**1.30 (1.07–1.59)**	**1.38 (1.10–1.73)**	**1.39 (1.12–1.72)**	**1.35 (1.09–1.66)**	**1.28 (1.00–1.64)**	1.20 (0.85–1.69)
17 Weeks	**1.17 (1.02–1.34)**	**1.31 (1.04–1.65)**	**1.39 (1.07–1.80)**	**1.40 (1.10–1.79)**	**1.36 (1.08–1.71)**	**1.29 (0.98–1.69)**	1.20 (0.83–1.74)
18 Weeks	**1.17 (1.00–1.38)**	**1.32 (1.01–1.73)**	**1.40 (1.04–1.89)**	**1.41 (1.06–1.87)**	**1.36 (1.05–1.78)**	1.29 (0.94–1.75)	1.20 (0.79–1.83)
19 Weeks	**1.18 (0.98–1.43)**	**1.33 (0.97–1.83)**	**1.41 (0.99–2.01)**	**1.42 (1.02–1.97)**	**1.37 (1.01–1.86)**	1.29 (0.90–1.84)	1.19 (0.73–1.96)
20 Weeks	1.19 (0.95–1.48)	1.34 (0.93–1.94)	**1.42 (0.95–2.14)**	**1.43 (0.97–2.09)**	**1.37 (0.96–1.96)**	1.29 (0.85–1.95)	1.19 (0.67–2.12)

Bold values highlight statistically significant (p < 0.05) results

Abbreviations: OR, odds ratio; CI, confidence interval

## Discussion

Rainfall is an important factor contributing to dengue incidence, in part due to mosquitoes’ reliance upon stagnant water pools to reproduce [7,31,32]. Excessive rainfall can flush out these breeding sites resulting in larvae death [33–35], potentially causing a reduction in dengue incidence in the following weeks.

To evaluate the relationship between excessive rainfall and flushing of larvae from their breeding sites, we developed the PLUM model to identify rainfall conditions that are associated with flushing events and to predict flushing occurrence. The PLUM model predicts daily flushing occurrence based upon the temporal rainfall pattern expressed through aggregation of excessively high cumulative and peak daily rainfall conditions. Our analysis has shown that a variety of temporal rainfall patterns is associated with flushing events. These patterns, highlighted in Fig 2, ranged from heavy rainfall directly preceding the flushing event to the accumulation of excess rainfall over several weeks followed by moderate rainfall directly preceding the flushing event. For example, one flushed observation exceeded 11 daily rainfall thresholds and three cumulative rainfall thresholds (indicating heavy rainfall preceding the event) while another flushed observation exceeded 3 daily rainfall thresholds and 19 cumulative rainfall thresholds (indicating the accumulation of excess rainfall over time followed by a smaller rainfall event that finally triggered flushing). Sensitivity analysis further emphasized the importance of the temporal rainfall pattern rather than single rainfall events. When using only one daily rainfall variable (e.g., peak daily total rainfall in the preceding two days) we observed a marked decrease in model accuracy and its ability to discriminate between flushed and non-flushed observations (Accuracy: 88%; AUC: 0.75) as compared to the PLUM model (Accuracy: 92%; AUC: 0.94; Table 2).

Flushing events were negatively associated with dengue outbreak risk in Singapore between 2000 and 2016 (Figs 3 and 4). This association was observed when five or more flushing events occurred in a week, and persisted up to six weeks after the flushing events occurred. The strongest association was found for weeks where flushing events occurred each day, reducing the risk of a dengue outbreak by as much as 70%. When there were five or six flushing events per week, the observed association was marginally attenuated (a maximum reduction of 48% and 56% respectively). These results provide support for the hypothesis put forth by Seidahmed and Eltahir that in addition to typical climatic, human, and vector drivers, monsoon-driven flushing events affect seasonal abundance of *Ae*. *aegypti* via a process of flushing and drying phases. Through this mechanism, excess rainfall flushes out *Aedes* larvae from the breeding site and subsequent dry periods impede *Aedes* breeding by preventing the development of adequate breeding sites [34]. Together, these flushing and drying periods can have a harmful effect on dengue transmission.

Our findings, that excess rainfall negatively affects dengue spread, are supported by multiple studies which have observed a similar inverse association between rainfall and mosquito borne disease incidence or dengue virus isolation [29,39,89,90]. For example, a study on the relationship between rainfall and malaria incidence in China found an inverse association (high rainfall was associated with a 15–50% reduction in malaria incidence) when incidence was modeled as a function of total rainfall at the 4^th^ and 6^th^, 9^th^, or 12^th^ lagged weeks, hinting at a similar flushing mechanism [90]. In Curaçao, monthly rainfall above 200mm, as compared with mean monthly rainfall at 54mm was associated with a decrease in dengue incidence [29], while in Colombo Sri Lanka, where annual rainfall (2.4m) is similar to Singapore (2.3m), weekly total rainfall was observed to have a weak negative association with dengue incidence [39]. In Senegal, annual dengue virus isolates from mosquitoes decreased 9.7% for each additional 1 in of rainfall from baseline [89].

In contrast, several studies have identified an increase in the risk of dengue associated with increasing rainfall. These reports used statistical models that assumed a linear relationship between rainfall and dengue incidence [19,30,37,39,48]. This approach may obscure a high non-linearity in the studied association, where a positive relationship is observed for low to moderate levels of rainfall and a negative relationship for high rainfall patterns. As excessively high rainfall patterns are rare as compared with more moderate and close to average rainfall patterns, linear models tend to reflect a strong positive relationship between rainfall and dengue incidence observed at low and moderate levels of rainfall [4,30]. Further, several of these and other studies characterized rainfall using a single variable (monthly or weekly average rainfall) [4,10,19,25–30,37,39]. Our analysis has shown that no single variable was sufficient to describe flushing and the associated reduction in dengue outbreak risk. The association was made visible through using a combination of variables reflecting the temporal rainfall pattern over several weeks.

Interestingly, we observed that when fewer than five flushing events occurred per week there was an increase in the risk of a dengue outbreak six to 19 weeks after the flushing events occurred. We believe this association is due to the increased rainfall associated with flushing, but not flushing itself. For example, with increased rainfall new breeding sites may be created, increasing *Ae*. *aegypti* abundance and subsequent dengue outbreak risk. This finding highlights the role of moderate rainfall in promoting dengue transmission which has been previously observed [19,30,37,39,48].

There are several limitations to the study. In developing the PLUM model, we could not account for the potential effect of El Niño Southern Oscillation because entomological data were obtained from a single year and the majority of flushed observations (88%) occurred during an El Niño period. Future studies should account for this possibility by analyzing entomological data from El Niño, La Niña, and neutral periods.

The PLUM model was developed using data obtained from 53 breeding sites from a single neighborhood and during a 12-month period. If the drainage thresholds for the roadside storm drains significantly varied across the time or location, PLUM model accuracy and generalizability would be negatively affected resulting in a biased model. Despite these potential limitations, we validated the generalization of the PLUM model on unseen data and found its performance to be robust (Table 2). Furthermore, the drainage conditions should be relatively invariant over time unless the storm drain is damaged or physically changed.

In the current study, we were only able to estimate the effect of the flushing out of breeding sites occurring in road side storm drains. There are other key-breeding sites (e.g., roof gutters, flower pots, and domestic containers) defined by the National Environmental Agency of Singapore that were not evaluated due to ethical and legal considerations [34]. Additional work is needed to evaluate how the flushing of other breeding habitats influence dengue spread.

We did not have information on vector control campaigns during the study period, an important confounding factor that is missing in our study. To compensate for its effect, we statistically adjusted the models for season, as vector control measures vary by season in Singapore.

The developed model associating flushing and dengue outbreak incidence relies on weekly flushing occurrence variable values, which have been inferred though the PLUM model. Even though the PLUM model achieved very high accuracy on the unseen test data (92% correct; Table 2), it still introduced misclassification error in defining days with flushing in the data, though we estimate the influence of such misclassification on the final results to be small.

As with any epidemiological data, the dengue incidence might have been significantly underreported. We assumed this bias be non-differential; at worst we would expect that the effect of underreporting would make results biased towards no association—indicating that the magnitude of the unbiased protective association would be stronger [91].

### Conclusions

We proposed a non-linear approach to understanding the relationship between excessive rainfall, flushing, and dengue outbreak occurrence in Singapore. According to the PLUM model, flushing conditions are characterized by rainfall patterns indicating excess rainfall. We demonstrated that rainfall-induced flushing is associated with a statistically significant decreased risk of dengue outbreak, with association being significant up to six weeks after the week when flushing occurred.

## Supporting information

S1 Fig The framework of the Predictive fLUshing-Mosquito (PLUM) model. The model identifies flushing events of the dengue mosquito *Ae*. *aegypti* using variables describing patterns of rainfall in the study areas and entomological data.
(TIF)

S1 Table UFA identified high and low risk thresholds. (DOCX)

S1 Text Supplemental methods and results. (DOCX)
