# Supplementary figures and images for: Statistical modeling of the effect of rainfall flushing on dengue transmission in Singapore
Source: PLoS Negl Trop Dis. 2018 Dec 6;12(12):e0006935. doi: 10.1371/journal.pntd.0006935 (PMC6283346; doi:10.1371/journal.pntd.0006935)

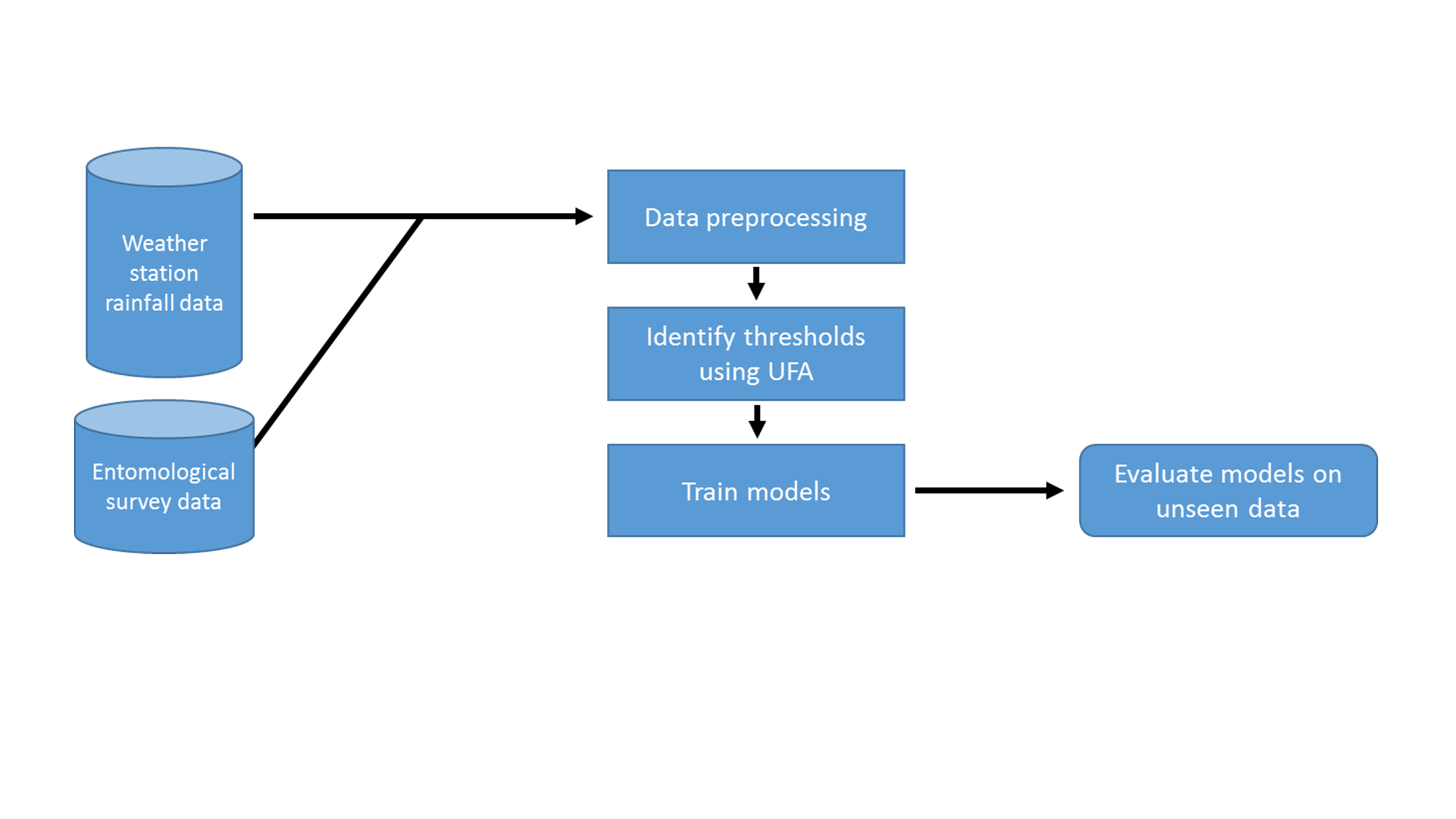

Supplement: S1 Fig — The model identifies flushing events of the dengue mosquito Ae. aegypti using variables describing patterns of rainfall in the study areas and entomological data. (TIF) [file pntd.0006935.s001.tif]
