# Supplementary material for: Statistical modeling of the effect of rainfall flushing on dengue transmission in Singapore
Source: PLoS Negl Trop Dis. 2018 Dec 6;12(12):e0006935. doi: 10.1371/journal.pntd.0006935 (PMC6283346; doi:10.1371/journal.pntd.0006935)
Supplement: S1 Table — (DOCX) [file pntd.0006935.s002.docx]

**S1 Table UFA identified high and low risk thresholds**

| **Variable** | **THRESHOLD** | **N** | **%Flushed** | **Risk Group** |
| --- | --- | --- | --- | --- |
| Average rainfall per rainy day (7-day period) | ≥ 13mm | 36 | 56% | High Risk |
| Average rainfall per rainy day (14-day period) | ≥ 16mm | 29 | 59% | High Risk |
| Average rainfall per rainy day (21-day period) | ≥ 14mm | 39 | 56% | High Risk |
| Average rainfall per rainy day (28-day period) | ≥ 15mm | 35 | 60% | High Risk |
| Cumulative rainfall (1-week period) | ≥ 55mm | 28 | 64% | High Risk |
| Cumulative rainfall (2-week period) | ≥ 123mm | 22 | 82% | High Risk |
| Cumulative rainfall (3-week period) | ≥ 210mm | 14 | 93% | High Risk |
| Cumulative rainfall (4-week period) | ≥ 242mm | 20 | 85% | High Risk |
| Cumulative rainfall (5-week period) | ≥ 316mm | 17 | 82% | High Risk |
| Cumulative rainfall (6-week period) | ≥ 341mm | 20 | 75% | High Risk |
| Cumulative rainfall (7-week period) | ≥ 452mm | 15 | 73% | High Risk |
| Cumulative rainfall (8-week period) | ≥ 622mm | 7 | 86% | High Risk |
| Cumulative rainfall (9-week period) | ≥ 736mm | 5 | 100% | High Risk |
| Cumulative rainfall (10-week period) | ≥ 444mm | 26 | 54% | High Risk |
| Cumulative rainfall (11-week period) | ≥ 399mm | 45 | 49% | High Risk |
| Cumulative rainfall (12-week period) | ≥ 493mm | 31 | 58% | High Risk |
| Cumulative rainfall (13-week period) | ≥ 529mm | 30 | 60% | High Risk |
| Cumulative rainfall (14-week period) | ≥ 544mm | 33 | 61% | High Risk |
| Cumulative rainfall (15-week period) | ≥ 568mm | 33 | 58% | High Risk |
| Cumulative rainfall (16-week period) | ≥ 663mm | 31 | 58% | High Risk |
| Cumulative rainfall (17-week period) | ≥740mm | 29 | 55% | High Risk |
| Cumulative rainfall (18-week period) | ≥1006mm | 8 | 75% | High Risk |
| Cumulative rainfall (19-week period) | ≥ 1028mm | 7 | 86% | High Risk |
| Cumulative rainfall (20-week period) | ≥ 1114mm | 6 | 83% | High Risk |
| Peak daily total rainfall (7-day period) | ≥ 47mm | 13 | 77% | High Risk |
| Second highest daily total rainfall (7-day period) | ≥ 23mm | 15 | 67% | High Risk |
| Third highest daily total rainfall (7-day period) | ≥ 19mm | 10 | 80% | High Risk |
| Fourth highest daily total rainfall (7-day period) | ≥ 2mm | 22 | 55% | High Risk |
| Fifth highest daily total rainfall (7-day period) | ≥ 2mm | 9 | 67% | High Risk |
| Sixth highest daily total rainfall (7-day period) | ≥ 1mm | 3 | 100% | High Risk |
| Peak daily total rainfall (1-day period) | ≥ 20mm | 9 | 89% | High Risk |
| Peak daily total rainfall (2-day period) | ≥ 20mm | 19 | 79% | High Risk |
| Peak daily total rainfall (3-day period) | ≥ 24mm | 26 | 65% | High Risk |
| Peak daily total rainfall (4-day period) | ≥ 47mm | 8 | 100% | High Risk |
| Peak daily total rainfall (5-day period) | ≥ 44mm | 14 | 86% | High Risk |
| Peak daily total rainfall (6-day period) | ≥ 47mm | 12 | 83% | High Risk |
