# Supplementary material for: Statistical modeling of the effect of rainfall flushing on dengue transmission in Singapore
Source: PLoS Negl Trop Dis. 2018 Dec 6;12(12):e0006935. doi: 10.1371/journal.pntd.0006935 (PMC6283346; doi:10.1371/journal.pntd.0006935)
Supplement: S1 Text — (DOCX) [file pntd.0006935.s003.docx]

# S1 Text Supplemental Methods and Results

# Materials and Methods

## PLUM model development

We used supervised machine learning models – Classification And Regression Trees (CART) [1], Random Forest (RF) [2], and Logistic Regression [3] to associate statistically significant thresholds with flushing. CART is a decision tree algorithm that recursively partitions the training data using binary splitting rules based on values of selected variables [4]. This continues until a splitting rule can no longer partition the data or the stopping criteria are met. RF is an ensemble extension of CART, repeatedly sampling variable subsets from the dataset with replacement. CART and RF models were implemented with the *rpart* and *randomForest* R packages [5,6].

The PLUM model was trained using a balanced training set where non-flushed observations were randomly under-sampled to generate a 1:1 ratio of flushed to non-flushed observations in the training set to avoid an algorithm classifying all observations as the majority class [71].

There are several approaches to integrate UFA identified thresholds into a multi-dimensional classifier. All approaches used for this study created a binary indicator variable for each statistically significant threshold that was identified. The indicator variable takes on the value of one if the value of the variable in observation exceeds the threshold and zero otherwise [7]. UFA-identified high risk thresholds were integrated with predictive models using three approaches:

1. Predictions are based upon the aggregate number of high risk thresholds met for each observation (Not used for RF because the model algorithm requires two or more variables to formulate predictions).
2. Predictions were based upon the aggregate number of high risk thresholds pertaining to cumulative rainfall (e.g., two-week cumulative rainfall) and daily rainfall (e.g., peak daily total rainfall in a seven-day period) variables met for each observation.
3. Predictions were based upon all high risk thresholds and the aggregate number of high risk thresholds pertaining to cumulative and daily rainfall variables. Because the logistic regression model was unable to accommodate all predictors, we used LASSO to select the variables to be used for prediction [8].

The model-approach combination with the best overall performance was selected as the final PLUM mode.

## PLUM model sensitivity analysis

In the entomological data set, multiple observations defined as flushed were found to have only one breeding site recorded as flushed and negative. The presence of one breeding site being classified as flushed and negative may indicate that another factor independent of rainfall may have caused the breeding site to be flushed. To assess this potential limitation, we performed a sensitivity analysis where we utilized two additional definitions to define a day of observation as *Flushed* or *Non-flushed*. The two alternative definitions user for the PLUM model outcome were:

1. We define a day as flushed if two or more breeding sites are classified as flushed and negative for aquatic stages. We define a day as non-flushed if fewer than two breeding sites were classified as flushed and negative for aquatic stages.
2. We define a day as flushed if two or more breeding sites are classified as flushed and negative aquatic stages. We define observations as not flushed if no breeding sites are classified as flushed and negative. Observations with one breeding site flushed and negative are removed from the analysis.

Using these two definitions, we employed the PLUM model framework described in Section 2.5 *Modeling and predicting flushing events* to predict flushing occurrence.

# Results

## PLUM model sensitivity analysis

In the sensitivity analysis, we identified five (20%) flushed observations where only one breeding site was classified as flushed and negative. Among these 5 observations, 4 occurred shortly after observations where at least 50% of observed breeding sites were classified as flushed and negative (Fig 1).

**Fig 1. Proportion of breeding sites flushed for each obtained entomological survey.** A) The number of breeding sites observed as flushed and negative over the course of the observation period. B) The number of breeding sites that were under observation for the day of interest. Among all observations, 82 (77%, red) were classified as not flushed and 25 (24%) were classified as flushed. Among all flushed observations, 20 (80%, blue) observations had two or more breeding sites classified as flushed and negative and 5 (80%, green) observations had one breeding site classified as flushed and negative. Of the 5 observations with only one breeding site classified as flushed and negative, 4 (80%) of these observations occurred within seven days after a flushing event where at least 50% of monitored breeding sites were classified as flushed and negative. Breeding sites were not monitored between February 21^st^ and March 10^th^.


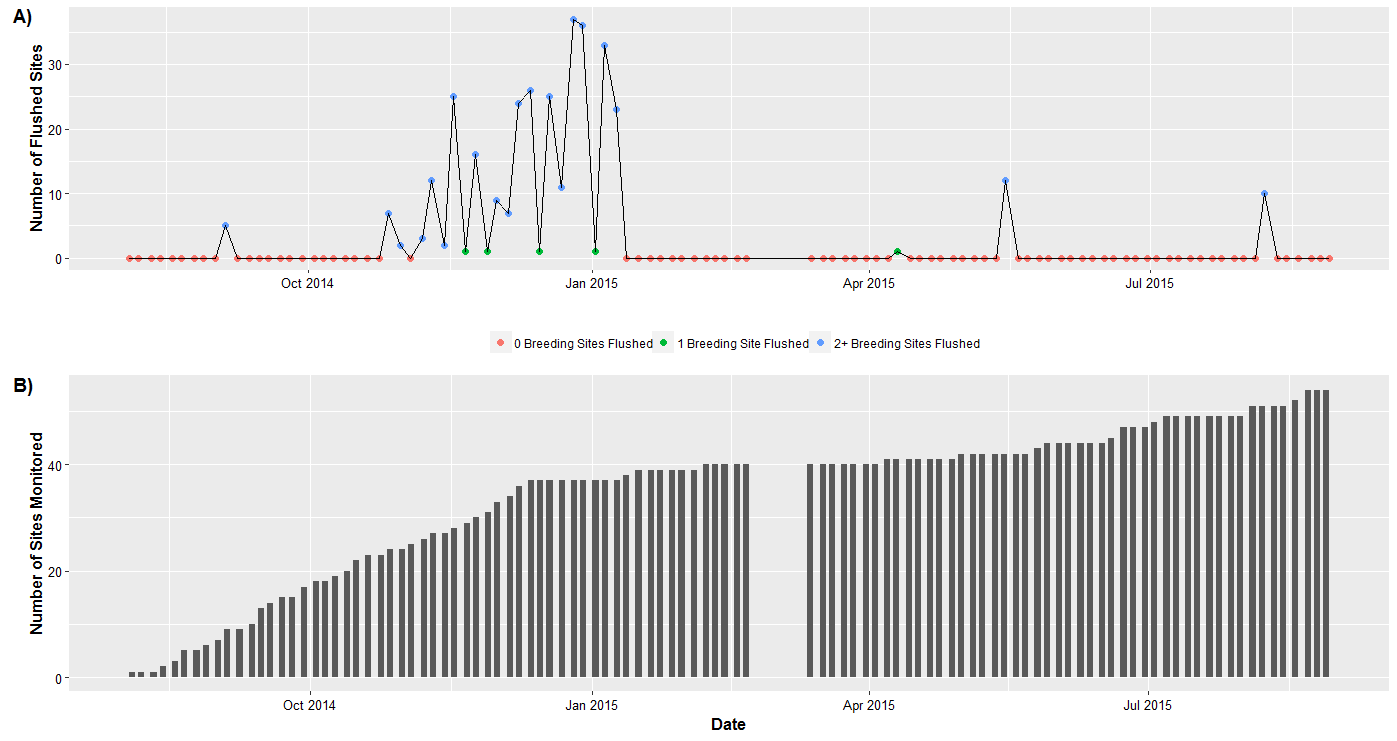


Twenty observations were defined as flushed based on both alternative flushed definitions (2+ or more breeding sites classified as flushed and negative). Eighty-seven observations were defined as not flushed based upon the first alternative not flushed definition (less than 2 breeding sites flushed) and 82 observations were defined as not flushed when using the second alternative not flushed definition (0 breeding sites flushed) and 5 observations were removed for not meeting the selection criteria.

### Sensitivity analysis of the rainfall conditions associated with flushing events

We applied UFA to identify thresholds associated with an increased likelihood of flushing occurrence. Running UFA for all 38 variables, we identified 37 high risk thresholds for both alternative outcome definitions (Table 1). Similar high risk thresholds were identified regardless of using the original (flushed observations: 1+ breeding site flushed and negative) or alternative outcome definitions.

**Table 1: High risk thresholds identified using original and alterative PLUM model outcome definitions.**

|  | **Original Outcome Definition** | | | **Alternative Outcome Definition 1** | | | | **Alternative Outcome Definition 2** | | |  |
| --- | --- | --- | --- | --- | --- | --- | --- | --- | --- | --- | --- |
| **Name** | **Threshold** | **N** | **% Flushed** | | **Threshold** | **N** | **% Flushed** | **Threshold** | **N** | **% Flushed** | **Risk Group** |
| Average rainfall per rainy day (7-day period) | ≥ 12.674 | 36 | 56% | | ≥ 14.218 | 30 | 50% | ≥ 14.4744 | 28 | 54% | High Risk Threshold |
| Average rainfall per rainy day (14-day period) | ≥ 15.685 | 29 | 59% | | ≥ 15.685 | 29 | 48% | ≥ 15.622 | 26 | 54% | High Risk Threshold |
| Average rainfall per rainy day (21-day period) | ≥ 13.136 | 39 | 56% | | ≥ 19.648 | 7 | 71% | ≥ 13.0697 | 37 | 51% | High Risk Threshold |
| Average rainfall per rainy day (28-day period) | ≥ 14.33 | 35 | 60% | | ≥ 19.135 | 4 | 100% | ≥ 19.1406 | 4 | 100% | High Risk Threshold |
| Cumulative rainfall (1-week period) | ≥ 54.832 | 28 | 64% | | ≥ 54.832 | 28 | 54% | ≥ 54.064 | 25 | 60% | High Risk Threshold |
| Cumulative rainfall (2-week period) | ≥ 122.64 | 22 | 82% | | ≥ 122.64 | 22 | 64% | ≥ 122.606 | 18 | 78% | High Risk Threshold |
| Cumulative rainfall (3-week period) | ≥ 209.892 | 14 | 93% | | ≥ 191.232 | 22 | 68% | ≥ 224.944 | 8 | 100% | High Risk Threshold |
| Cumulative rainfall (4-week period) | ≥ 241.9 | 20 | 85% | | ≥ 307.264 | 10 | 80% | ≥ 241.936 | 16 | 81% | High Risk Threshold |
| Cumulative rainfall (5-week period) | ≥ 316 | 17 | 82% | | ≥ 385.12 | 9 | 78% | ≥ 317.122 | 14 | 79% | High Risk Threshold |
| Cumulative rainfall (6-week period) | ≥ 340.208 | 20 | 75% | | ≥ 340.208 | 20 | 60% | ≥ 337.802 | 17 | 71% | High Risk Threshold |
| Cumulative rainfall (7-week period) | ≥ 451.152 | 15 | 73% | | ≥ 602.688 | 4 | 100% | ≥ 573.44 | 4 | 100% | High Risk Threshold |
| Cumulative rainfall (8-week period) | ≥ 621.504 | 7 | 86% | | ≥ 680.096 | 3 | 100% | ≥ 617.672 | 6 | 83% | High Risk Threshold |
| Cumulative rainfall (9-week period) | ≥ 735.4 | 5 | 100% | | ≥ 743.712 | 3 | 100% | ≥ 739.718 | 3 | 100% | High Risk Threshold |
| Cumulative rainfall (10-week period) | ≥ 443.072 | 26 | 54% | | ≥ 702.68 | 9 | 56% | ≥ 447.248 | 23 | 48% | High Risk Threshold |
| Cumulative rainfall (11-week period) | ≥ 398.544 | 45 | 49% | | ≥ 398.544 | 45 | 40% | ≥ 395.472 | 42 | 43% | High Risk Threshold |
| Cumulative rainfall (12-week period) | ≥ 493 | 31 | 58% | | ≥ 501.84 | 27 | 48% | ≥ 496.692 | 26 | 54% | High Risk Threshold |
| Cumulative rainfall (13-week period) | ≥ 528.16 | 30 | 60% | | ≥ 528.16 | 30 | 47% | ≥ 521.52 | 26 | 54% | High Risk Threshold |
| Cumulative rainfall (14-week period) | ≥ 543.236 | 33 | 61% | | ≥ 543.236 | 33 | 48% | ≥ 539.71 | 29 | 55% | High Risk Threshold |
| Cumulative rainfall (15-week period) | ≥ 567.26 | 33 | 58% | | ≥ 567.26 | 33 | 45% | ≥ 569.224 | 29 | 52% | High Risk Threshold |
| Cumulative rainfall (16-week period) | ≥ 662.852 | 31 | 58% | | ≥ 580.812 | 39 | 44% | ≥ 659.152 | 27 | 52% | High Risk Threshold |
| Cumulative rainfall (17-week period) | ≥ 739.72 | 29 | 55% | | ≥ 608.584 | 44 | 41% | ≥ 611.2 | 40 | 45% | High Risk Threshold |
| Cumulative rainfall (18-week period) | ≥ 1005.82 | 8 | 75% | | ≥ 1005.82 | 8 | 63% | ≥ 1005.606 | 7 | 71% | High Risk Threshold |
| Cumulative rainfall (19-week period) | ≥ 1027.368 | 7 | 86% | | ≥ 1027.368 | 7 | 71% | ≥ 1027.5 | 6 | 83% | High Risk Threshold |
| Cumulative rainfall (20-week period) | ≥ 1113.312 | 6 | 83% | | ≥ 1113.312 | 6 | 67% | ≥ 1115.12 | 5 | 80% | High Risk Threshold |
| Peak daily total rainfall (7-day period) | ≥ 46.872 | 13 | 77% | | ≥ 46.872 | 13 | 69% | ≥ 52.752 | 9 | 78% | High Risk Threshold |
| Second highest daily total rainfall (7-day period) | ≥ 22.112 | 15 | 67% | | ≥ 22.112 | 15 | 53% | ≥ 22.088 | 13 | 62% | High Risk Threshold |
| Third highest daily total rainfall (7-day period) | ≥ 18.028 | 10 | 80% | | ≥ 18.028 | 10 | 60% | ≥ 18.006 | 8 | 75% | High Risk Threshold |
| Fourth highest daily total rainfall (7-day period) | ≥ 1.76 | 22 | 55% | | ≥ 4.048 | 13 | 54% | ≥ 4.048 | 12 | 58% | High Risk Threshold |
| Fifth highest daily total rainfall (7-day period) | ≥ 1.44 | 9 | 67% | | ≥ 1.824 | 6 | 67% | ≥ 1.44 | 8 | 63% | High Risk Threshold |
| Sixth highest daily total rainfall (7-day period) | ≥ 0.408 | 3 | 100% | | ≥ 0.408 | 3 | 100% | ≥ 0.408 | 3 | 100% | High Risk Threshold |
| Peak daily total rainfall (1-day period) | ≥ 19.372 | 9 | 89% | | ≥ 19.372 | 9 | 89% | ≥ 19.372 | 9 | 89% | High Risk Threshold |
| Peak daily total rainfall (2-day period) | ≥ 19.2 | 19 | 79% | | ≥ 28.6 | 11 | 91% | ≥ 28.6 | 11 | 91% | High Risk Threshold |
| Peak daily total rainfall (3-day period) | ≥ 23.32 | 26 | 65% | | ≥ 46.54 | 5 | 100% | ≥ 46.544 | 5 | 100% | High Risk Threshold |
| Peak daily total rainfall (4-day period) | ≥ 46.8 | 8 | 100% | | ≥ 46.8 | 8 | 100% | ≥ 46.76 | 9 | 89% | High Risk Threshold |
| Peak daily total rainfall (5-day period) | ≥ 43.776 | 14 | 86% | | ≥ 43.776 | 14 | 79% | ≥ 47.41 | 7 | 100% | High Risk Threshold |
| Peak daily total rainfall (6-day period) | ≥ 46.528 | 12 | 83% | | ≥ 46.528 | 12 | 75% | ≥ 46.49 | 11 | 82% | High Risk Threshold |
| Number of rainy days (7-day period) | NA | NA | NA | | ≥ 6.06 | 4 | 75% | ≥ 6.06 | 4 | 75% | High Risk Threshold |

Original outcome definition: Flushed observations 1+ breeding site flushed and negative; Not flushed observations: 0 breeding sites flushed and negative
Alternative outcome definition 1: Flushed observations 2+ breeding site flushed and negative; Not flushed observations: < 2 breeding sites flushed and negative
Alternative outcome definition 2: Flushed observations 2+ breeding site flushed and negative; Not flushed observations: 0 breeding sites flushed and negative

### Final PLUM model selection

We integrated UFA-identified thresholds into CART, RF, and logistic regression models using three approaches (*section 1.1* in S1 Text). Based upon the data, Logistic regression and RF demonstrated similar performance. Given that Logistic regression is a less complex model, it was selected as the final PLUM model over RF. We found that the most predictive variables were the aggregate number of high risk thresholds that were met per day for both cumulative and daily rainfall variables (Fig. 2).

**Fig 2. Performance of all candidate PLUM model’s classification on the unseen test data.** Predictive accuracy was optimized using the logistic regression model and when predictions were based upon the aggregate number of high risk thresholds that were met per day for both cumulative and daily rainfall variables. Evaluation measures include accuracy, F1-score, positive predictive value, Sensitivity, area under the receiver operating characteristics curve (AUC), specificity, and negative predictive value.


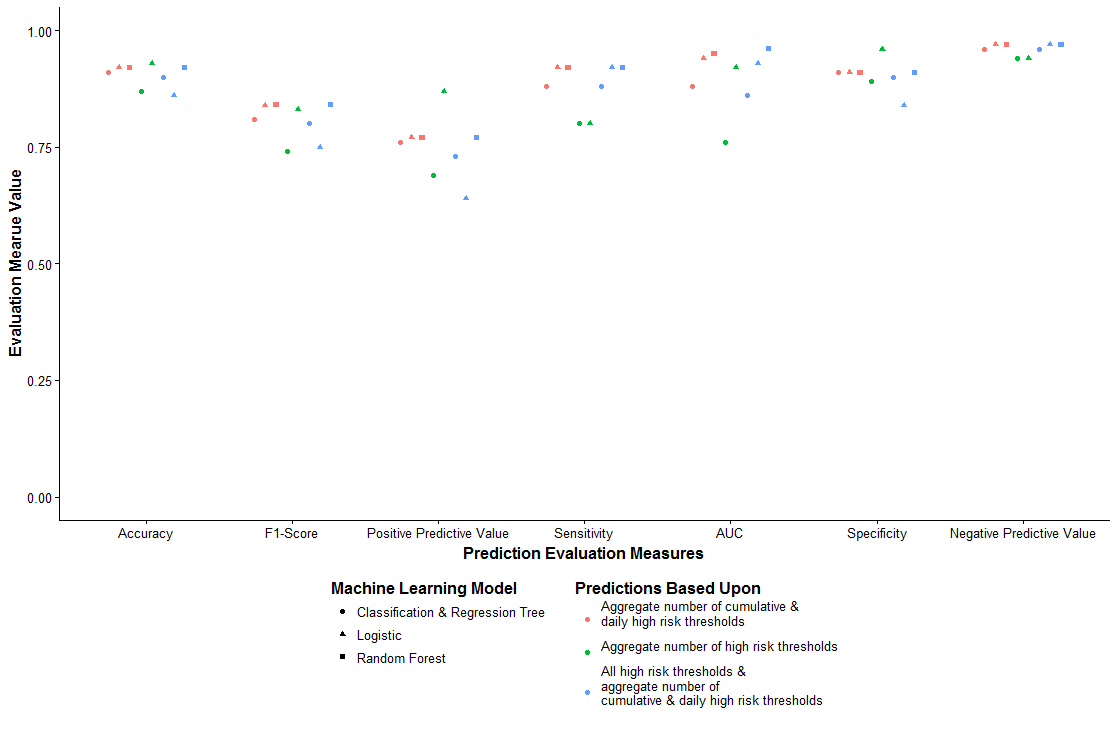


### Sensitivity analysis of predicting flushing events from rainfall pattern

For both alternative definitions, we assessed PLUM model performance where predictions were based upon the number of cumulative and daily high risk thresholds met (Table 2). When using the first alternative outcome definition, model performance decreased in terms of accuracy (-6%), F1-score (-0.13), positive predictive value (-0.19), and specificity (-0.06). When using the second alternative outcome definition, model performance remained unchanged. These results are most likely due to the fact that four of the five observations that had only one flushed breeding site occurred shortly after observations where several breeding sites were flushed where the observations have similar preceding rainfall patterns. Given the similar rainfall patterns, the PLUM model misclassified these observations as flushed (when using the first alternative outcome definition) which would degrade model performance.

**Table 2 Analysis of PLUM model sensitivity to flushing definition**

| **Outcome Definition** | **Model** | **Predictor Variables** | **Acc** | **F1** | **PPV** | **Se** | **AUC** | **Sp** | **NPV** |
| --- | --- | --- | --- | --- | --- | --- | --- | --- | --- |
| Original outcome definition | Logistic | # cumulative rainfall high risk thresholds  # daily rainfall high risk thresholds | 92% | 0.84 | 0.77 | 0.92 | 0.94 | 0.91 | 0.97 |
| Alternative outcome definition 1 | Logistic | # cumulative rainfall high risk thresholds  # daily rainfall high risk thresholds | 86% | 0.71 | 0.58 | 0.90 | 0.94 | 0.85 | 0.97 |
| Alternative outcome definition 2 | Logistic | # cumulative rainfall high risk thresholds  # daily rainfall high risk thresholds | 92% | 0.83 | 0.73 | 0.95 | 0.95 | 0.91 | 0.99 |

Abbreviations: Acc, accuracy; F1, F1-score; PPV, Positive predictive value; Se, Sensitivity; AUC, Area under the receiver operating characteristic curve; Sp, Specificity; NPV, Negative predictive value
Original outcome definition: Flushed observations defined as 1+ breeding site flushed and negative; Not flushed observations defined as 0 breeding sites flushed and negative
Alternative outcome definition 1: Flushed observations defined as 2+ breeding site flushed and negative; Not flushed observations defined as < 2 breeding sites flushed and negative
Alternative outcome definition 2: Flushed observations defined as 2+ breeding site flushed and negative; Not flushed observations defined as 0 breeding sites flushed and negative

# S1 Text References

1. Breiman L, Friedman J, Stone CJ, Olshen RA. Classification and regression trees. CRC press; 1984.

2. Breiman L. Random forests. Mach Learn. 2001;45: 5–32.

3. Hosmer Jr DW, Lemeshow S, Sturdivant RX. Applied logistic regression [Internet]. John Wiley & Sons; 2013. Available: https://books.google.com/books?hl=en&lr=&id=64JYAwAAQBAJ&oi=fnd&pg=PA313&dq=logistic+regression&ots=DrkM8VcolL&sig=5NIZmzyVi_HdVaI7FFeMULXTNTU

4. Stingone JA, Pandey OP, Claudio L, Pandey G. Using machine learning to identify air pollution exposure profiles associated with early cognitive skills among U.S. children. Environ Pollut. 2017;230: 730–740. doi:10.1016/j.envpol.2017.07.023

5. Therneau T, Atkinson B, Ripley B. rpart: Recursive Partitioning and Regression Trees. R package version 4.1–10. 2015.

6. Liaw A, Wiener M. Classification and regression by randomForest. R News. 2002;2: 18–22.

7. Sheth M. Predicting mortality for patients in critical care: a univariate flagging approach. Massachusetts Institute of Technology. 2015.

8. Muthukrishnan R, Rohini R. LASSO: A feature selection technique in predictive modeling for machine learning. Advances in Computer Applications (ICACA), IEEE International Conference on. IEEE; 2016. pp. 18–20.
